# Unconventional interplay between heterovalent dopant elements: Switch-and-modulator band-gap engineering in (Y, Co)-Codoped CeO_2_ nanocrystals

**DOI:** 10.1038/srep15415

**Published:** 2015-10-21

**Authors:** T. S. Wu, H. D. Li, Y. W. Chen, S. F. Chen, Y. S. Su, C. H. Chu, C. W. Pao, J. F. Lee, C. H. Lai, H. T. Jeng, S. L. Chang, Y. L. Soo

**Affiliations:** 1 Department of Physics, National Tsing Hua University, Hsinchu, Taiwan; 2 Department of Materials Science and Engineering, National Tsing Hua University, Hsinchu, Taiwan; 3 National Synchrotron Radiation Research Center, Hsinchu, Taiwan; 4 Institute of Physics, Academia Sinica, Taipei, Taiwan

## Abstract

We report the experimental observation and theoretical explanation of an unconventional interplay between divalent Co and trivalent Y dopants, both of which incur oxygen vacancies in the CeO_2_ host that has predominantly tetravalent Ce cations. The Co dopant atoms were experimentally found to act as a switch that turns on the dormant effect of Y-modulated band-gap reduction. As revealed by density functional theory (DFT) calculations with structures verified by synchrotron-radiation x-ray measurements, a Co 3d band that hybridizes with Ce 4f band was lowered due to reduced O 2p repulsion arising from oxygen vacancies incurred by Y doping and therefore gave rise to the observed band-gap narrowing effect. Such switch-and-modulator scheme for band-gap engineering in nanocrystal materials can lead to important applications in environmental protection and solar energy harvesting technologies.

Controllable modulation of energy band gaps in crystalline materials can lead to novel applications in a wide variety of technologies ranging from catalysis, photovoltaics to optical and electronic devices. We have attempted to modulate the band gap of CeO_2_ (ceria) nanocrystals by incorporating heterovalent dopant elements into the material. Nanostructures of ceria are widely used in the development of solar cells and environmental protection catalysts^1^,^2^,^3^. Oxygen vacancies in the form of minority trivalent cerium in CeO_2_, where Ce are predominantly tetravalent, play an important role in affecting the electronic structures and magnetism of ceria^4^,^5^,^6^. The catalytic activity of nanostructured CeO_2_ catalysts has also been found to increase substantially with the oxygen vacancy concentration due to vacancy-induced electronic structural changes^2^. Therefore, the electronic properties of CeO_2_ may presumably be manipulated by defect engineering techniques that alter the oxygen vacancy concentration, such as doping impurity atoms with valences less than 4+. In fact, a previous report has shown that doping of trivalent Nd can indeed lead to non-monotonic band-gap changes in ceria nanoparticles^7^.

In this work, we chemically incorporated trivalent Y and divalent Co dopant ions into ceria nanocrystals attempting to modulate the band gap of the doped host. Presumably, the only major effect of divalent Co ions in trivalent-Y-ion-doped ceria may simply be generating more oxygen vacancies and therefore enhance the existing effects incurred by yttrium. However, contrary to the above reasoning, our experimental measurements have shown that the divalent Co dopant atoms actually play a much more subtle role, namely a switch that turns on the dormant Y-modulated band-gap variation in Y-doped CeO_2_. To understand the mechanism of such unconventional interplay between Y and Co dopants, the crystal structure of the nanocrystal CeO_2_ host and the local structures surrounding the Y and Co dopant atoms were precisely determined by synchrotron radiation x-ray techniques. First-principle theoretical calculations were then carried out to obtain electronic structures of the codoped nanocrystal system with initial guess of structural model suggested by the x-ray analyses.

A polyol method was used to prepare the samples of Y-doped and (Y, Co)-codoped CeO_2_ nanocrystals. A slurry of Y(NO_3_)_3_.6H_2_O, CoCl_2_.6H_2_O and Ce(NO_3_)_3_.6H_2_O in diethylene glycol was vigorously stirred and heated to 160 °C. After 1 ml NH_4_OH was injected, the solution was continually heated for 120 minutes at 160 °C under reflux. The resulting reacting mixture was cooled to room temperature to form a suspension. Finally, a yellow precipitate was collected from the suspension by centrifugation, repeatedly washed with a mixture of deionized water and ethanol, and then dried in an oven to form a powder sample. The Y and Co dopant concentrations were determined by inductively coupled plasma mass spectrometry (ICPMS). For the (Y, Co)-codoped samples, A1, A2, A3, A4 and A5, the Y concentrations are 0, 3.6, 7.1, 13.4, and 18.7 at.%, respectively, while the Co concentrations are about 4 at.%. For samples doped with Y only, B1, B2, B3, B4 and B5, the Y concentrations are 0, 3.3, 6.3, 12.2, and 16.7 at.%, respectively.

To determine the long-range-order crystal structures, x-ray powder diffraction (XRD) measurements were carried out for all samples using synchrotron radiation. As shown in Fig. 1, the CeO_2_ (111), (200), (220), (311), (222) and (400) Bragg peaks appear in the XRD patterns indicating cubic CeO_2_ structure for all samples. The lattice constant of the dopant-free sample 5.4160 Å is close to the bulk-ceria value 5.4111 Å. Due to the much smaller ion radius of Co than that of Ce, the lattice constant of the sample doped with Co only is reduced to 5.4001 Å. When Y is incorporated into the samples, the lattice constant decreases monotonically with increasing Y concentration from 5.4001 Å and 5.4160 Å for samples A1 and B1 to 5.3920 Å and 5.4114 Å for samples A5 and B5 in the Y-doped and (Y, Co)-codoped series, respectively. Such Y-induced lattice contraction may arise from the increase of oxygen vacancy concentration due to Y doping as demonstrated in previous reports^3^,^8^. The crystallite sizes were determined from XRD by using the Scherrer equation^9^ to be around 4–5 nm for all samples.

Local structures surrounding Y and Co dopant atoms in the CeO_2_ host were probed by using the extended x-ray absorption fine structure (EXAFS) technique at beamlines BL07A and BL17C of National Synchrotron Radiation Research Center (NSRRC) in Taiwan. Conventional fluorescence mode was adopted using a Lytle fluorescence detector for Y K-edge and Co K-edge absorption measurements^10^,^11^. An established data reduction method was used to extract χ functions from the raw experimental data^12^. The Fourier transforms and EXAFS χ functions for the Y K-edge and Co K-edge EXAFS are plotted as fine lines in Figs 2 and 3, respectively. Local structural parameters were extracted using a curve-fitting procedure based on the FEFF software^10^,^13^. The amplitude reduction factor S_0_^2^ representing the central atom shake-up and shake-off effects and the mean free path of photoelectrons λ were set to be 0.9 and 10 Å for the Y data and 0.68 and 10 Å for the Co data as determined from curve-fitting the EXAFS data for Y_2_O_3_ and CoO model compounds, respectively^11^,^14^. The curve-fittings exhibit a O shell at 2.31 ± 0.01 Å and a Ce shell at 3.77 ± 0.01 Å from the Y central atom. In comparison to the Ce-O distance of 2.34 Å and Ce-Ce distance of 3.83 Å of CeO_2_, the observed Y-O and Y-Ce distances indicate that the Y dopant atoms most likely occupy Ce sites in the CeO_2_ matrix. However, the coordination number of the nearest O shell surrounding Y is around 6.0 ± 0.5 to 7.0 ± 0.6, roughly one less than that of 8 surrounding Ce in the CeO_2_ host. The reduced coordination number of the nearest O shell indicates that an oxygen vacancy is generated around the Y dopant atom. The Co EXAFS data show only the first O shell at distances around 2.05 ± 0.01–2.07 ± 0.01 Å surrounding the Co atoms, which is very different from the Ce-O bond length. The coordination number of the nearest O shell surrounding Co is around 5.2 ± 0.4 to 5.8 ± 0.1, roughly two less than that surrounding Ce. This indicates that Co atoms may occupy Ce sites and are surrounded by around two oxygen vacancies that give rise to large distortion. As a consequence of the large distortion, short-range order was not observed by EXAFS beyond the nearest neighboring shell.

To determine the effective valencies of Y and Co in the samples, the Y and Co K-edge near-edge x-ray absorption fine structure (XANES) spectra for the doped ceria samples are plotted with those of model compounds of different Y or Co valencies in Fig. 4. As demonstrated in the insets, the XANES spectra for all samples are very similar to each other at both the Y and Co K edges indicating similar local bonding environment surrounding Y and Co in all samples, respectively. By comparing the edge position of the samples with those of model compounds, it is clear that the effective valencies for Y and Co in the ceria samples are 3+ and 2+, respectively. Therefore, it is confirmed by the XANES data that trivalent Y and divalent Co dopants were incorporated in the CeO_2_ host of tetravalent Ce.

The band gap variation due to Y and Co doping was monitored by UV-vis diffuse reflectance spectra as shown in Fig. 5. The band gap energies, associated with O 2p to Ce 4f transition, are determined by fitting the absorption data with Kubelka-Munk function^15^ and Tauc’s plots^16^ to be 2.74 eV, 2.58 eV, 2.43 eV, 2.31 eV, and 2.28 eV, for the (Y, Co) codoped CeO_2_ samples, A1, A2, A3, A4, and A5, respectively. The band gap energies for samples doped with Y only, B1, B2, B3, B4, and B5, are 2.80 eV, 2.83 eV, 2.81 eV, 2.84 eV, and 2.80 eV, respectively. The band gap of Co-free Y-doped CeO_2_ appears to remain largely unchanged as the Y concentration increases from 0 to 16.7%. However, when Co codopant of concentration 4% is added to the Y-doped CeO_2_, the band gap decreases monotonically from 2.74 eV to 2.28 eV as the Y concentration increases from 0 to 18.7%. As shown in the inset of Fig. 5, the Co and Y dopants act as a switch and a modulator for band gap tuning, respectively. It is worth noting that, as the band gap decreases with increasing Y concentration in the (Y, Co)-codoped samples, the lattice parameter also decreases monotonically as indicated by the XRD data (Fig. 1). Therefore, the possible mechanism adopted by previous reports that lattice relaxation leads to band-gap reduction is not applicable to our case^7^,^17^. As a matter of fact, the increase of Y concentration, while increasing oxygen vacancy concentration according to our EXAFS analysis, does not substantially reduce the band gap in the absence of the Co codopant.

To understand the observed switch-and-modulator effect, first-principle electronic structures calculations based on the density functional theory (DFT) were performed using the Vienna *ab initio* simulation package (VASP)^18^ with an initial guess of structural model suggested by the x-ray analyses. In our calculations, a generalized gradient approximation (GGA) PBEsol functional^19^ was used. Compared to other methods, the GGA PBEsol+U method can better reproduce structural parameters and therefore are more suitable for our purpose^20^,^21^,^22^. A Hubbard U correction (GGA+U) to the Ce 4f orbitals with an effective U of 6.5 eV was employed to describe the correlation effects of the Ce 4f states in defective CeO_2_ crystals. A 2 × 2 × 2 supercell of the cubic Fm
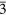
m fluorite structure with 96 atoms therein was used as the initial model for pure CeO_2_. The Brillouin-zone (BZ) integration was performed using a 3 × 3 × 3 Monkhorst- Pack k-point grid^23^. The plane-wave kinetic energy cutoff was set to be 400 eV^20^. Atomic positions and lattice parameters of all models were optimized without any symmetry restrictions until the maximum force on each atom was smaller than 0.025 eV/Å. To mimic oxygen vacancies, O atoms were removed from the pure CeO_2_ supercell. For the sample doped with Y, a Ce atom next to an oxygen vacancy was replaced by a Y atom. For the sample doped with Co, a Co atom substitutes for a Ce atom, where two O neighboring atoms are removed from the diagonal sites of a CoO8 cube. Finally, the (Y, Co)-codoped CeO_2_ model was constructed with Co at the center and Y on the edges of the supercell (Fig. 6). The dependence of band gap on Y concentration was investigated by comparing the result of one Y atom per supercell (3.1 at.%) to that of two Y atoms per supercell (6.2 at.%). The concentration of Co was one Co atom per supercell (3.1 at.%). After structural relaxation and stabilization, the calculated lattice constant 5.3929–5.4107 Å are close to the experimental values 5.3920–5.4160 Å. The calculated average Y-O and Co-O bond lengths are 2.298 Å and 2.00 Å, which are close to the experimental values of 2.31 ± 0.01 Å and 2.05 ± 0.01–2.07 ± 0.01 Å from EXAFS analysis, respectively. Since the calculated structures agree well with the x-ray results, the calculated densities of states (Fig. 7) are expected to be highly reliable.

The calculated O 2p to Ce 4f band gap for pure CeO_2_ (2.53 eV) is ~0.3 eV smaller than the experimental value of 2.80 eV. This is expected since GGA is well-known to underestimate band gap values. When an oxygen vacancy is introduced, a peak representing the f states localized around the two Ce ions nearest to the O vacancy appears in the middle of the band gap without changing the gap value (Fig. 7b). Doping with Y alone does not change the general shape of the density of states (Fig. 7c). Doping with Co alone can introduce two Co 3d impurity bands; one is in the middle of the band gap and the other hybridizes with Ce 4f orbitals right below the conduction band edge (Fig. 7d). While the O 2p to Ce 4f band gap remains unchanged if only one of the two dopants is incorporated, substantial band gap narrowing occurs when both Y and Co are present. At the Y concentrations of 3.1 at.% and 6.2 at.%, the calculated band gap is reduced from 2.53 eV of the pure ceria to 2.23 eV (Fig. 7e) and 2.17 eV (Fig. 7f), respectively. In comparison, the experimental band gap dropped from 2.74 eV to 2.58 eV and 2.43 eV as Y concentration increases from 0 at.% to 3.6 at.% and 7.1 at.%, respectively. Except for the underestimated band gap values of the GGA method, our calculations well reproduced the general trend of the Co-triggered and Y-modulated band gap variations observed experimentally. From the partial density of states plotted in Fig. 7, it is obvious that the Co 3d band right below the Ce 4f band edge plays a pivotal role in the observed Y-modulated band gap narrowing effect. As the Y concentration is increased, more oxygen vacancies are generated. Therefore, the repulsion of O 2p on Co 3d is lowered and the Co 3d band shifts to a lower energy. Since such Co 3d band largely hybridizes with the Ce 4f band, the Ce 4f band edge is lowered as a result of Y doping. Subsequently, the O 2p to Ce 4f band gap decreases with increasing Y concentration in the (Y, Co)-codoped CeO_2_.

Our results show that increasing the oxygen vacancy concentration by Y doping alone cannot modulate the band gap in the oxygen-vacancy-sensitive CeO_2_ nanocrystals. However, band gap narrowing via Y-induced oxygen vacancies can be achieved by incorporating a small amount of a triggering dopant element that generates an impurity band in the gap and largely hybridizes with the conduction band. In the present work, Co acts as a switch while Y plays the role of a modulator for tuning the band gap in the CeO_2_ host. The interplay of such switch and modulator codopants for band gap engineering can lead to many important applications, especially in environmental protection and solar energy harvesting.

## Additional Information

**How to cite this article**: Wu, T. S. *et al*. Unconventional interplay between heterovalent dopant elements: Switch-and-modulator band-gap engineering in (Y, Co)-Codoped CeO_2_ nanocrystals. *Sci. Rep.* **5**, 15415; doi: 10.1038/srep15415 (2015).

## Figures and Tables

**Figure 1:**
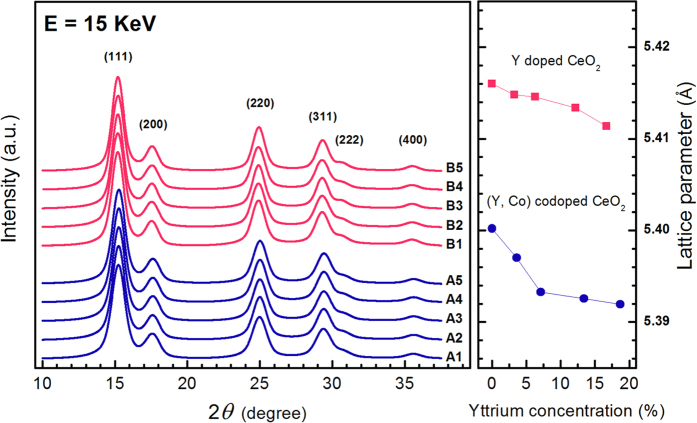
X-ray powder diffraction patterns and lattice parameter vs. Y concentration curves for the Y doped (upper) and the (Y, Co) codoped (lower) samples. Curves have been shifted vertically for the sake of clarity.

**Figure 2:**
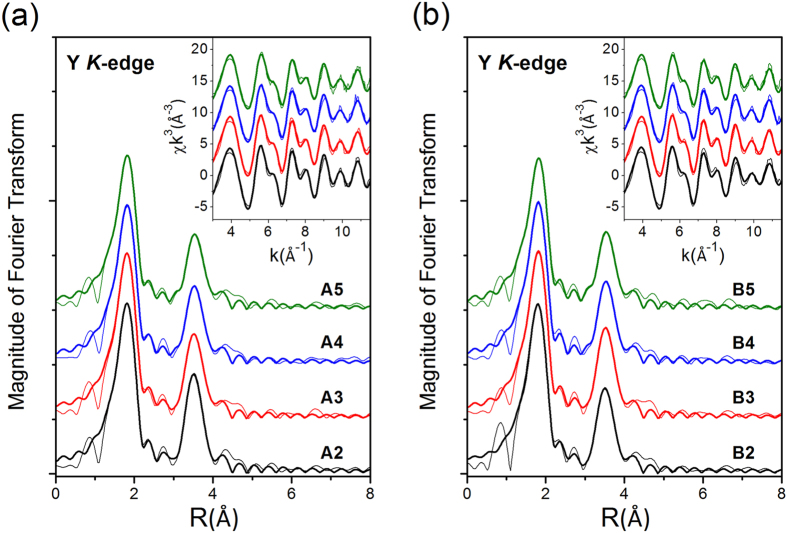
(**a**) Y K-edge EXAFS data for (Y, Co) codoped CeO_2_ samples. (**b**) Y K-edge EXAFS data for Y doped CeO_2_ samples. Fine lines: experimental; Coarse lines: curve fitting. Curves have been shifted vertically for the sake of clarity.

**Figure 3:**
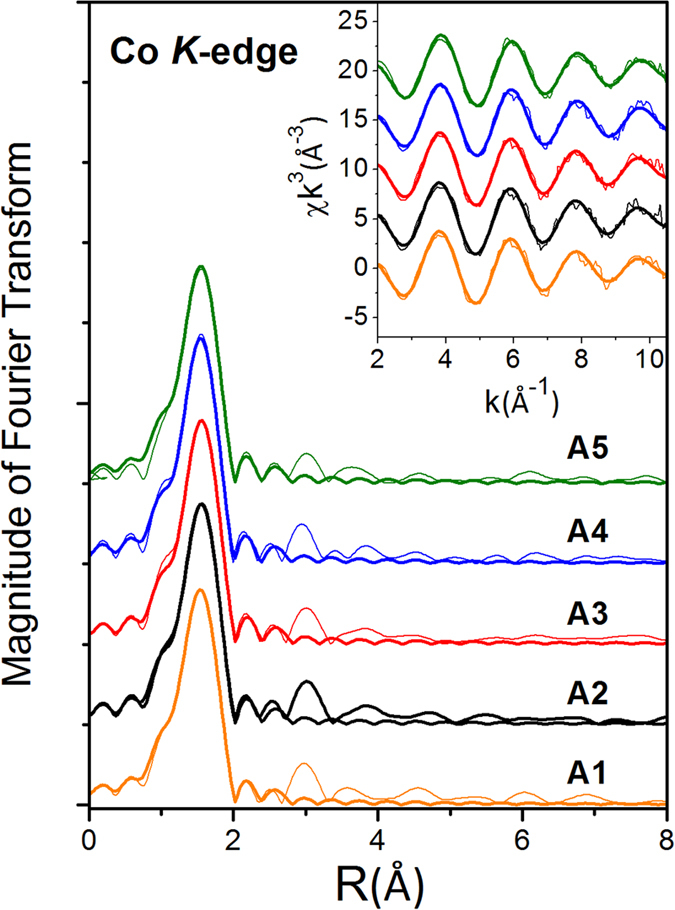
Co K-edge EXAFS data for (Y, Co) codoped CeO_2_ samples.

**Figure 4:**
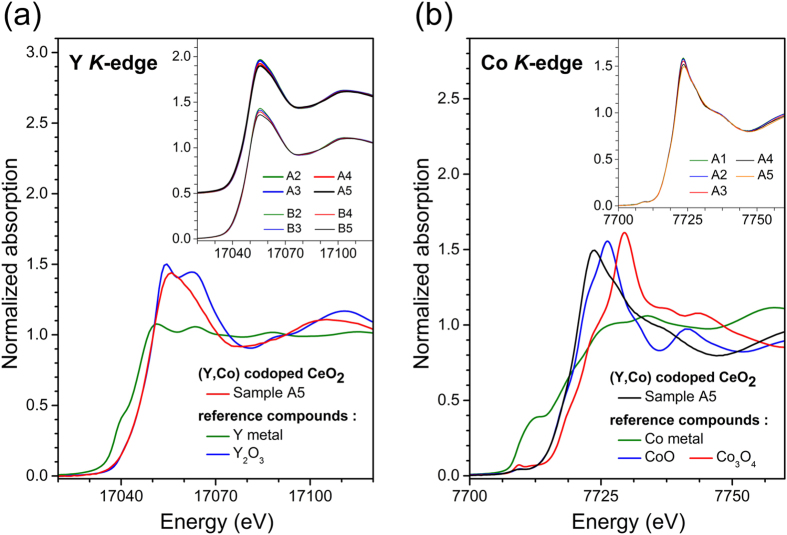
(**a**) Y K-edge XANES data for Y doped and (Y, Co) codoped CeO_2_ samples. (**b**) Co K-edge XANES data for (Y, Co) codoped CeO_2_ samples.

**Figure 5:**
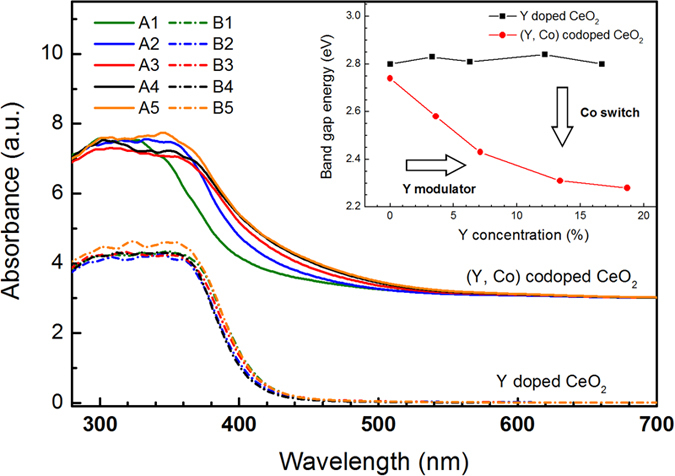
(**a**) UV–vis diffuse reflectance spectra for the Y doped (lower) and the (Y, Co) codoped (upper) samples. Inset: A plot of band gap energy vs. Y concentration.

**Figure 6:**
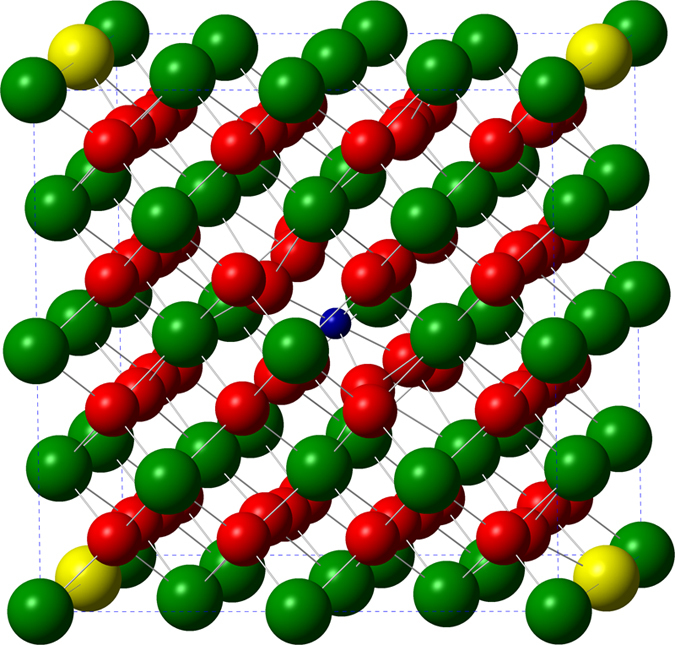
Schematic view of (Y, Co) codoped CeO_2_ structural models. The large red balls, large green balls, large yellow balls and small blue balls represent the O, Ce, Y and Co atoms, respectively.

**Figure 7:**
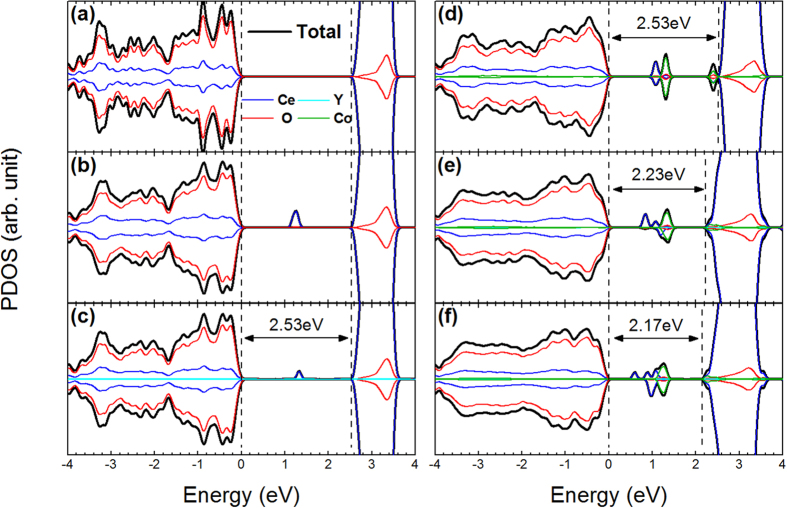
Total and partial densities of states for (**a**) Pure CeO_2_, (**b**) oxygen vacancy (V_O_) doped CeO_2_, (**c**) Y-doped CeO_2_, (**d**) Co-doped CeO_2_ and (**e**) (Y, Co)-codoped CeO_2_ with 3.1 at.% of Y and (**f**) (Y, Co)-codoped CeO_2_ with 6.2 at.% of Y. To clearly compare the band gaps, O 2p band edge of all structures were aligned in the plot.
